# Data-driven direct diagnosis of Li-ion batteries connected to photovoltaics

**DOI:** 10.1038/s41467-023-38895-7

**Published:** 2023-05-30

**Authors:** Matthieu Dubarry, Nahuel Costa, Dax Matthews

**Affiliations:** 1 Hawaiʻi Natural Energy Institute, University of Hawaiʻi at Mānoa, 1680 East West Road, POST 109, Honolulu, HI 96822 USA; 2 Computer Science Department, Polytechnic School of Engineering, University of Oviedo, Gijon, 33202 Asturias Spain

**Keywords:** Batteries, Computer science, Electrical and electronic engineering, Solar cells, Energy grids and networks

## Abstract

Photovoltaics supply a growing share of power to the electric grid worldwide. To mitigate resource intermittency issues, these systems are increasingly being paired with electrochemical energy storage devices, e.g., Li-ion batteries, for which ensuring long and safe operation is critical. However, in this operation framework, secondary Li-ion batteries undergo sporadic usage, which prevents the application of standard diagnostic methods. Here, we propose a diagnostic methodology that uses machine learning algorithms trained directly on data obtained from photovoltaic charging of Li-ion batteries. The training is carried out on synthetic voltage data at various degradation conditions calculated from clear sky model irradiance data. The method is validated using synthetic voltage responses calculated from plane of array irradiance observations for a photovoltaic system located in Maui, HI, USA. We report an average root mean square error of 2.75% obtained for more than 10,000 different degradation paths with 25% or less degradation on the Li-ion cells.

## Abstract (web summary)

Li-ion batteries are used to store energy harvested from photovoltaics. However, battery use is sporadic and standard diagnostic methods cannot be applied. Here, the authors propose a methodology for diagnosing photovoltaics-connected Li-ion batteries that use trained machine learning algorithms.

## Introduction

In recent years solar photovoltaic (PV) technologies provided the most additional generating capacity to the United States grid^1^. In 2021, a record 23.6 GW of solar capacity was installed, and over the next 10 years, it is predicted that 324 GW of new solar capacity will be added to the electric grid, quadrupling current levels^1^. Solar energy harvesting systems are increasingly being paired with secondary electrochemical energy storage systems, like Li-ion batteries (LiBs), at multiple grid levels. While some of the storage will be performed by grid-scale batteries, the percentage of residential storage installations has also been steadily increasing, reaching 8.1% in 2020^2^. It is estimated that by 2025, one in three residential solar systems will be paired with small-scale energy storage^1^, most likely powered by LiBs^3^.

To ensure long, safe, and continuous operation, LiBs must be maintained and controlled properly, which includes the regular estimation of their state of health (SOH). Current state-of-the-art SOH estimation methods can be found in recent reviews^4,5^, but their application could be problematic for LiBs paired with PV because of the sporadic usage in both charge and discharge. As a result of this unpredictability, the diagnosis might only be performable under lengthy maintenance cycles. An alternative to avoid downtime could be to identify and take advantage of auspicious conditions to perform state estimation. With LiBs supposed to last a decade or more, there are opportunities for different approaches, such as using batteries response under clear sky conditions under which PV power production is predictable for up to 12 h^6^.

Even if the PV power output offered by clear sky conditions is predictable, state estimation will still be complex and require robust methodologies for LiB diagnosis. Because the batteries paired with PV will not be operated under constant current (CC), the standard features^7^ to estimate SOH might be difficult to interpret. This favors data-driven methods, and in particular, machine learning (ML) methods^8^. However, to be applicable, ML algorithms need to be trained on a wide variety of data covering the sporadicity of the application. Unfortunately, while PV-connected lead acid batteries data are reported in the literature^8^, and some market data are already accessible^9,10^, no data for LiBs are yet available. Few studies are available on LiBs testing associated with PV duty cycles^11,12^, with most of the studies being modeling-centered and using CC testing^13–19^. Looking at CC data, the lack-of-data problem was recently solved with the introduction of synthetic datasets that enabled the emulation of every possible battery degradation^20–23^. While the duty cycle for clear sky irradiance will be more complex, recent work suggests that the methodology used to generate the synthetic data could be applied outside of CC^24^ and thus be applicable to irradiance.

**Fig. 1:**
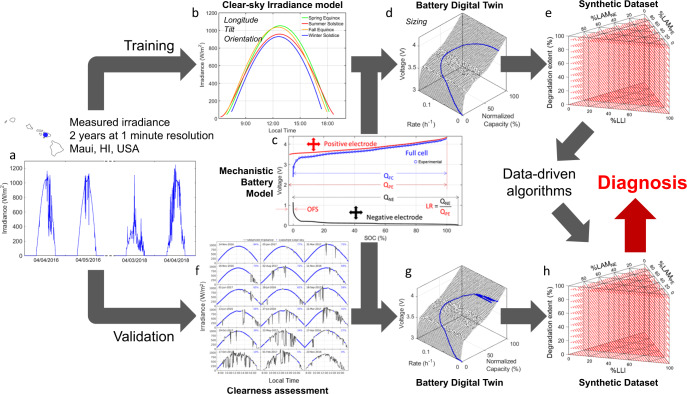
Schematic representation of the proposed approach. **a** Example of measured irradiance data from the test site in Maui, HI, USA. **b** Clear sky simulations at equinoxes and solstices (spring equinox in green, summer solstice in red, fall equinox in orange, and winter solstice in blue). **c** Digital twin calibration (positive electrode in red, negative electrode in black, full cell in blue, and experimental data in blue circles). **d** Li-ion cell simulation using the digital twin for clear skies. **e** Map of the tested degradation modes combinations. **f** Clear sky percentage assessment with real irradiances in black and detected clear sky conditions in blue. **g** Li-ion cell simulation using the digital twin for cloudy skies. **h** Map of the tested degradation modes combinations.

In this work, we propose a method for diagnosing PV-connected batteries using synthetic datasets that would allow for SOH estimation during normal operations. The method uses periods of clear sky conditions, where charging from PV generation is relatively stable and predictable, for diagnosis. We also report a framework for (1) generating synthetic datasets of the voltage response of Li-ion cells charged by PV systems, (2) synthetic dataset training of state-of-the-art ML algorithms, and (3) algorithm validation using synthesized data. The framework, which consists of a branch for training and a branch for validation, is summarized in Fig. 1.

The training branch uses irradiance data from a clear sky model (CSM), PV system information (longitude, tilt, orientation), and LiB information (chemistry and power) to generate synthetic cycles consisting of the voltage response of the cells for specific duty cycles under tens of thousands of different degradations. This dataset is then used to train, test, and compare selected ML algorithms for diagnosis. The selection includes a Random Forest regressor (RF)^25^, an extreme gradient boosting regressor (XGB)^26^, a feed-forward neural network (FNN)^27^, a 1-dimensional convolutional neural network (1D-Conv)^20^, and a dynamic time warping 2-D convolutional neural network (DTW)-CNN^28^.

While the validation would ideally be performed on data from deployed LiBs, no deployed PV-linked LiBs dataset is publicly available to the best of our knowledge. Even if data were available, the actual degradation of each individual system would not likely be, making validation implausible. In anticipation of data becoming available, we examined the applicability of our approach to real systems by replacing the deployed data with synthetic datasets generated for various sky-clearness levels, Fig. 1. These synthetic datasets are used to validate the applicability of the clear sky irradiance trained ML algorithms for diagnosis under cloudy conditions. To further emulate realistic conditions, each dataset was calculated on a Li-ion cell with slightly different parameters to account for cell-to-cell variations and inhomogeneities^29^. A selection of the data generated from this work is available in data repositories^30,31^.

## Results

### Irradiance data selection

The output of a PV system is dependent on irradiance, which is the power of the solar radiation striking the panels. Irradiance variability is driven by extraterrestrial and atmospheric effects and is also dependent on panel orientation^32,33^. The latter has become more varied in recent years^3^, but panels are nominally oriented toward the equator at a tilt angle near the latitude of the installation in order to maximize solar energy yield^34^.

Clear sky irradiance occurs during clear sky conditions, defined as an absence of visible clouds across the entire sky dome^6^. Clear sky irradiance is estimated using a CSM, which calculates solar geometry and accounts for variations in air mass and variations in optical depth^35^. In this work, we used the CSM proposed by Ineichen and Perez^35^ for a horizontal surface extended to estimate clear sky irradiance on a tilted surface in the plane of array (POA) of a PV panel. The extended CSM recomputes the solar angle of incidence, accounts for the reduction of diffuse irradiance received^36^, and adds a new ground-reflected irradiance source^37^. The accuracy of the CSM is primarily dependent on the accuracy of the broadband turbidity factor (TL) model input^38^. In this study, TL data was sourced from monthly mean climatological values extracted from the Solar Radiation Data Service^39^. Over the 2-year dataset used in this work, the CSM reproduced observed irradiance values with a relative root mean square error (RMSE) under 4%, with an *R*^2^ value above 0.99.

**Fig. 2:**
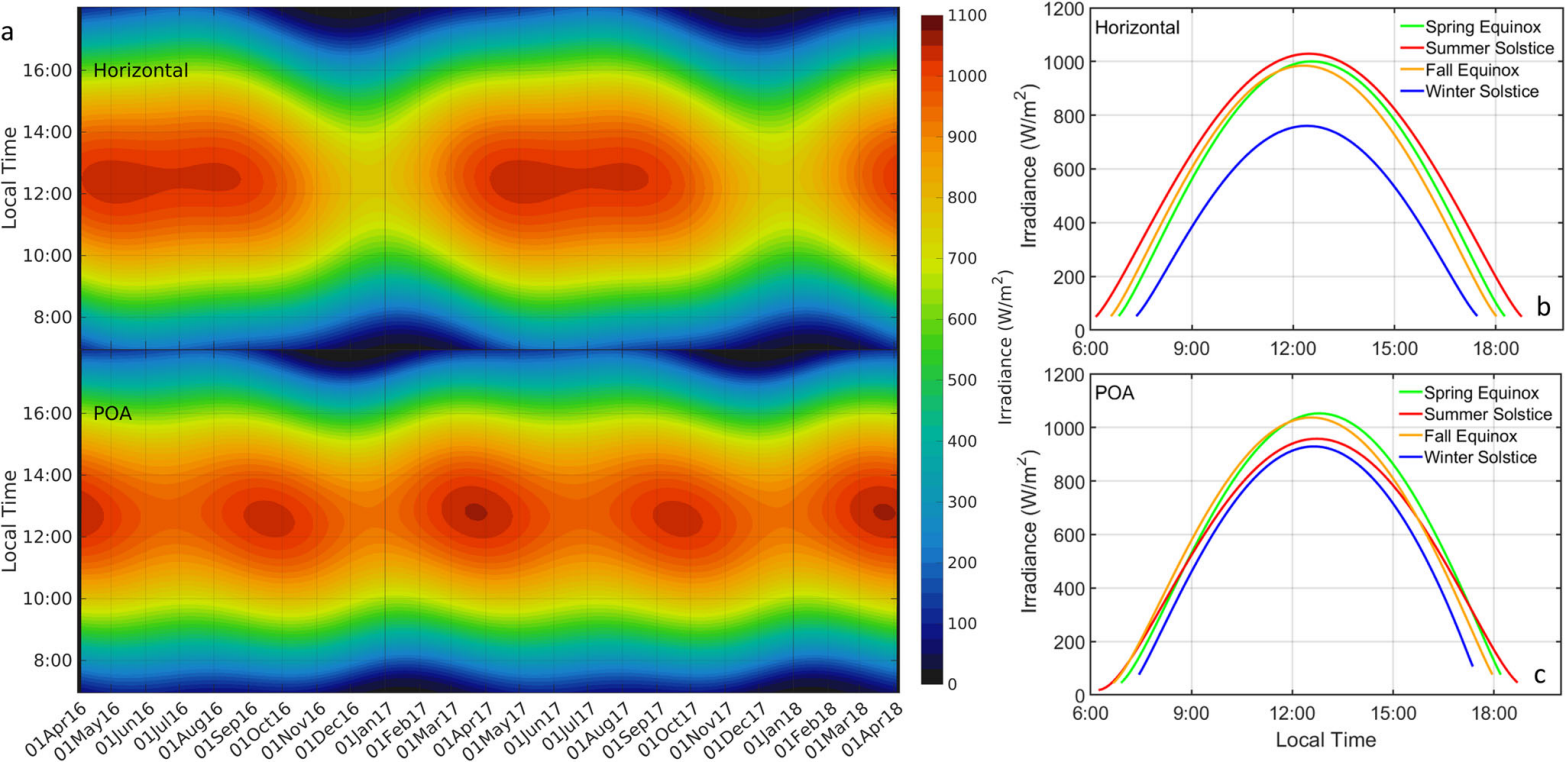
Clear sky irradiance Simulations. **a** Diurnal and seasonal variations in clear sky irradiance from a CSM for the test site in Maui, HI, USA. **b** Horizontal and **c** POA irradiances at equinoxes and solstices (spring equinox in green, summer solstice in red, fall equinox in orange, and winter solstice in blue).

Figure 2a presents diurnal and seasonal variations in horizontal (top) and POA (bottom) clear sky irradiance estimated by the CSM over a 2-year period for a PV test site located at the Maui Economic Development Board (MEDB) office on the southwestern coast of the island of Maui, Hawaiʻi, USA, with PV panels oriented at a 20° tilt with a 197° N azimuth. Due to Hawaiʻi’s proximity to the Northern Tropic, clear sky irradiance levels incident on a horizontal surface remained high (>900 W/m^2^) from April to September, with a small drop during winter (by 250 W/m^2^). Seasonal variations of clear sky irradiance incident on the POA of PV panels located at the site are reduced by half, with peak levels above 1000 W/m^2^ found in the spring and summer months. POA irradiance also peaks later in the day (by around 1 h) relative to horizontal values due to the panels facing slightly westward instead of due south. To further illustrate seasonal fluctuations, data at solstice and equinoxes are presented in Fig. 2b, c for horizontal and POA irradiances, respectively.

**Fig. 3:**
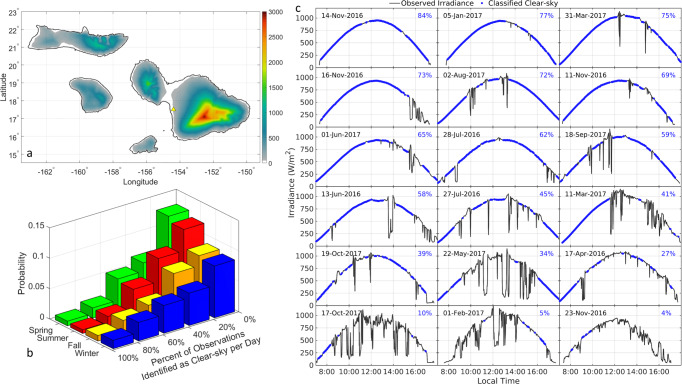
Irradiance measurements summary. **a** Maui topographic map with system location. **b** Seasonal distribution of clear sky distributions (spring in green, summer in red, fall in orange, and winter in blue). **c** Selected cloudy days irradiances (black) with associated clear sky periods (blue).

The CSM does not account for several factors driving real irradiance variability. While the most obvious is cloud cover, fluctuations in atmospheric turbidity, shading, soiling, and reflection losses also affect the amount of irradiance available for a PV system^40^. In this work, clear sky conditions were identified using an algorithm that applies a series of threshold criteria tests to compare the smoothness, shape, and magnitude of observed values within a moving window to corresponding clear sky values from the CSM^41,42^. The algorithm assigns a daily clearness value, determined using the number of observations identified as the clear sky over the total number of observations during daytime conditions. This value could be seen as an inverse noise indicator, 100% being no noise and 0% only noise, as cloud coverage will disrupt the theoretical output of the PV system with up and down irradiance spikes. The distribution of daily clearness for the Maui location, Fig. 3a, and per season for the whole dataset is shown in Fig. 3b. For almost half of the 2-year dataset, clear sky conditions were found in less than 20% of the daily observations, however, in nearly one in 5 days, more than 50% of observations were identified as clear sky. Moreover, the distribution of daily clearness values indicates only slight seasonal variations at the test site.

To assess how the accuracy of the diagnosis was affected by irradiance variability, 18 days from the 2-year dataset were selected to encompass a range of irradiance conditions, Fig. 3c. The conditions range from a minimum clearness of 4% to a maximum of 84%, with cloud cover occurring at contrasting times. Cloud effects range from small perturbations, likely due to high cirrus clouds, to significant attenuation and cloud enhancement, due to more opaque cloud cover. Shading effects caused by the construction of a nearby building can also be seen in the afternoon hours of the two days in October 2017.

### Cell emulation and duty cycle emulation

**Fig. 4:**
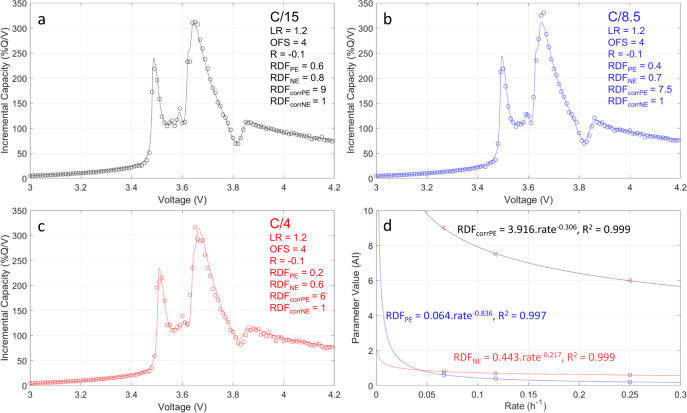
Graphite | |lithium nickel manganese cobalt Li-ion cell emulation from lab-scale Li metal cells. Li-ion cell emulation for different current rates with associated parameters: **a** C/15, **b** C/8.5, **c** C/4. **d** Variations of RDF_PE_ (blue), RDF_NE_ (red), and RDF_corrPE_ (black) with the current rate.

As presented in Fig. 1, a digital twin was used to generate the LiB data needed to assess the impact of the different duty cycles on battery performance. The LiB model included in the twin was based on the ‘alawa mechanistic model^43,44^. To parameterize the model and emulate the electrochemical response of the selected commercial LiB, the data for both the positive and negative electrodes (PE and NE, respectively), obtained from testing cells equipped with Li metal counter electrode, were imported into the ‘alawa toolbox. The Li metal cell data was fitted to the Li-ion cell by scanning different values for the loading ratio (LR), offset (OFS), resistance (R), and rate degradation factor (RDF) for the PE and the NE. Because the duty cycles simulated in this work were not CC, the ‘alawa model needed to correctly simulate current rates within the range used by the duty cycles of LiBs paired with PV. This calibration required emulations at different current rates and verification of continuity between the rate-dependent emulation parameters to enable interpolation and extrapolation to other rates. Figure 4a–c presents the results of the full-cell emulation of the C/15, C/8.5, and C/4 cycles, respectively, based on the Li metal cell data gathered from the harvested electrodes (a C/1 rate corresponds to a full charge in 1 h). The best fit had an LR of 1.2 with a 4% offset and a −0.1 resistance correction for the current rate-independent parameters. Looking at the rate-dependent parameters, the RDFs for both electrodes were found to decrease from 0.6 to 0.2 for the PE and from 0.8 to 0.6 for the NE as the simulated rate increased. An additional resistance correction was needed to compensate for peak movements for the RDF_PE_ (RDF_corrPE_). This correction ensured that the electrochemical response at different current rates overlapped correctly when kinetics was adjusted, which cannot currently be done automatically by the model. The equation for this additional resistance correction is provided in Supplementary Fig. 1 with an explanatory schematic. No correction was needed for the RDF_NE_. The evolution of the three varying rate-dependent parameters could be fitted with power laws with *R*^2^ ≥ 0.997 vs. *R*^2^ ~ 0.97 for linear regressions, Fig. 4d.

**Fig. 5:**
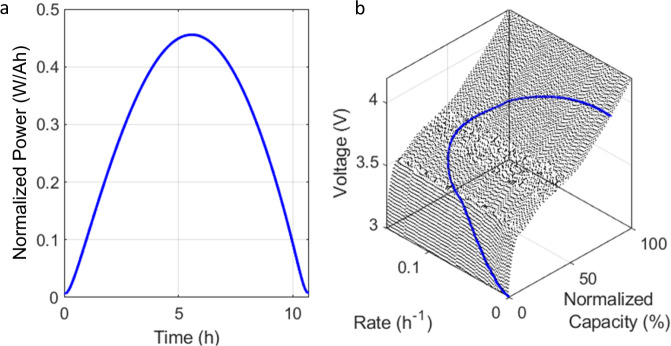
Photovoltaic Duty Cycle Emulation. **a** Example of an ideal clear sky PV power generation. **b** Associated voltage and rate variations versus SOC for graphite | |lithium nickel manganese cobalt Li-ion cell to match the power input (in blue) from a fully discharged state.

Using the best-fit parameters and equations, the synthetic Li-ion cell voltage response under the different duty cycles was generated by applying the method proposed in reference^24^ using solar panel power output as a duty cycle instead of CC. An example of clear sky solar panel power output is presented in Fig. 5a. The power is 0 at sunrise, ramps up to its maximum around solar noon, then ramps down to 0 at sunset. As proposed in ref. ^24^, and in order to simulate this duty cycle, a set of 100 voltage responses were simulated between the lowest current rate (minimum power at maximum voltage) and the highest current rate (maximum power at minimum voltage), Fig. 5b. The correct [voltage, current rate] couple to match the required power was calculated for each 0.1% state of charge until full charge. Overall, the maximum rate was chosen to be C/6 so that around 95% of the cell capacity is used through an average day (spring equinox, March 21st). C/6 is below the highest rate for which the emulation parameters were deciphered (C/4). This will allow high confidence in the simulation of high loss of active material (LAM) because, with at most 50% degradation, the local rate would, at worst double from C/6 to C/3^24,43^, which is still close to the range of experimentally tested rates.

Thermodynamic LiB degradation can be grouped into three degradation modes, the loss of lithium inventory (LLI) and LAM on both the PE and the NE^24,43^, because independent of what mechanism is inducing degradation, what will change is how much of each electrode is available to host lithium and how many lithium ions are able to go back and forth. Each combination of LLI, LAM_PE_, and LAM_NE_ corresponds to a unique degradation and has a unique voltage signature. Diagnosis of a Li-ion battery then corresponds to the quantification of the three degradation modes. As proposed in the literature^21–23^, the different degradations were simulated by scanning the entire range of possible combinations for LLI and LAMs. Once generated, the data were used to train and validate ML algorithms. More details on the synthetic data generation and the training are provided in the method section.

**Fig. 6:**
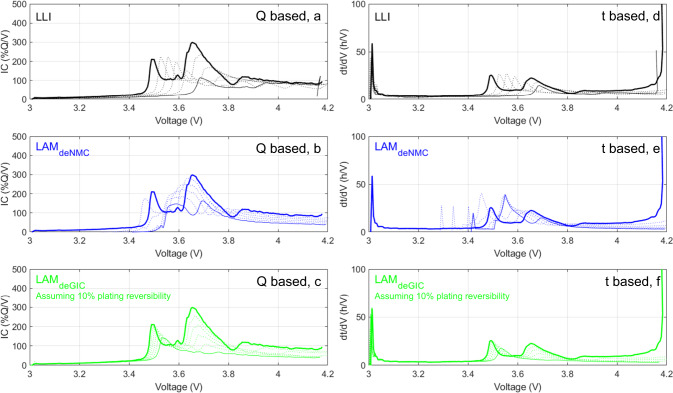
Graphite | |lithium nickel manganese cobalt degradation modes impact on the Li-ion cell voltage response. IC and IT degradation maps for the synthetic duty cycles generated from the perfect horizontal irradiance data at the MEDB site on the spring equinox for up to 50% degradation (pristine Li-ion cell: thick line, aged Li-ion cell: thin line, dotted lines: 10% increments in degradation). **a** *Q*-based LLI. **b** *Q*-based LAM_PE_. **c** *Q*-based LAM_NE_. **d** *t*-based LLI. **e** *t*-based LAM_PE_. **f** *t*-based LAM_NE_.

All the selected ML algorithms used in this work were developed to use features from a derivative of the voltage response, such as incremental capacity (IC, d*Q*/d*V* = *f*(*V*)), under CC as input. IC curves analysis is well known to facilitate the analysis of the changes in the voltage response of LiBs^45^ and thus diagnosis. In order to determine if these algorithms could be applied to irradiance duty cycles, it was necessary to verify that the associated derivative voltage response still showcased the expected features. Figure 6a–c presents simulations of the voltage response of a Li-ion cell, plotted as IC, for individual degradation modes as calculated using the ‘alawa model from the clear sky irradiance on the spring equinox. This degradation map is useful for assessing the impact of degradation on the voltage response. The voltage evolutions in Fig. 6a–c closely resemble the one observed for a conventional graphite | |lithium nickel manganese cobalt oxides cell tested under CC^21,44^. This provides confidence that the diagnosis algorithms developed under CC can be used on the data generated from PV irradiance.

Since the simulations were not performed under CC, the voltage response versus time is different than the voltage response versus capacity. This is because capacity corresponds to time multiplied by current; capacity and time are thus only directly correlated if the current is constant. The time vs. voltage data offers a different dataset that could be available for training and validation if features are identifiable. Figure 6d–f presents the t-based equivalent to the IC degradation maps (IT, d*t*/d*V* = *f*(*V*)). Despite some deformations, the *t*-based curves showcase significant similarities to their capacity counterparts and are, therefore, also well suited for degradation mode quantification using the selected algorithms. In this work, both the capacity (*Q*) and time (*t*) based datasets were generated and analyzed to determine if a *t*-based method could be as accurate as a *Q*-based one.

### Diagnosability

Three sets of experiments were performed in this research work. More details can be found in the method section. Training for the first two sets of experiments was performed on synthetic data generated from clear sky irradiance for the spring equinox. The spring equinox was selected because its POA clear sky irradiance is close to the yearly average. For the initial set of experiments representing an ideal case, validation was performed using the same data as the training. For the second set of experiments aimed at quantifying the impact of seasonal variability on diagnosis accuracy, validation was performed using synthetic data generated from clear sky irradiance for the first day of each month. Finally, for the third set of experiments, to test the impact of cloud cover, training was performed on synthetic data generated from clear sky irradiance for the 18 cloudy days detailed in the “Irradiance data selection” section with validation using synthetic data generated from observed irradiance for the same days.

To assess if the ML algorithms trained on clear sky irradiance were able to diagnose different battery degradations, they were first validated using the same clear sky irradiance. The only difference between the training and validation datasets was the cell parameters that were slightly varied to take cell-to-cell variations into consideration (cells 1 and 2 in Supplementary Table 1, see methods for more details). The first 4 rows of Table 1 present the average RMSE between the real and predicted values for more than 100,000 different combinations of the three degradation modes. The algorithms were all able to quantify each degradation mode properly with RMSEs of 2.1% at worst. Since smaller RMSEs were observed for lower degradations, Supplementary Fig. 2a–e, Table 1 presents the average RMSEs for the *Q*- and *t*-diagnosis for degradations with at most 25% and 50% of each degradation mode. Statistics for the full dataset with additional metrics such as the mean absolute error (MAE) and Pearson’s correlation coefficient (*ρ*) are provided in Supplementary Table 2. Overall, RMSEs below 0.85% for 25% or less degradation and 1.66% for 50% or less degradation were observed. Looking at the Q-diagnosis (top two rows), all algorithms performed nearly identically with average RMSEs around 0.70% for 25% or less degradation and 1.5% for 50% or less degradation. The average RMSEs of t-based data (rows 3 and 4) were similar, but the individual algorithm performance varied. XGB, FNN, and 1DConv showed similar RMSEs, 1DConv showed a lower average RMSE at 0.37%, and DTW-CNN RMSE nearly doubled compared to its *Q*-based counterpart. From the complete statistics in Supplementary Table 2, LLI seems the easiest to diagnose before LAM_PE_ for *Q*-based diagnosis, while the opposite holds true for *t*-based ones. In both cases, LAM_NE_ was the hardest to decipher. In general, all the calculated RMSEs were small, below or near 2.1% at worst, for more than 100,000 tested degradations up to 50% degradation, demonstrating that data from irradiance duty cycles can be successfully diagnosed for the ideal case of a single day with no cloud coverage at all.

**Table 1:** RMSE summary statistics for diagnosis from perfect irradiance data for the spring equinox (top four rows, sample size = 100,000) and for every 1st day of the month (bottom four rows, sample size = 43,000)

	RF	XGB	FNN	1DConv	DTW-CNN
*Q*-based, same day, <25% deg.	1.09	0.70	0.62	0.50	0.49
*Q*-based, same day, <50% deg.	2.08	1.88	1.15	1.15	1.10
*t*-based, same day, <25% deg.	1.27	0.81	0.64	0.45	1.00
*t*-based, same day, <50% deg.	2.11	1.96	1.64	1.08	1.51
*Q*-based, different day, <25% deg.	1.53	1.24	0.84	0.66	0.79
*Q*-based, different day, <50% deg.	2.80	3.15	1.71	1.73	1.53
*t*-based, different day, <25% deg.	3.30	3.24	3.56	3.25	5.75
*t*-based, different day, <50% deg.	4.43	4.42	5.43	4.45	7.62

Associated full statistics are available in Supplementary Table 2.

In the baseline case presented above, training and validation were performed on the same clear sky data. The second set of experiments was used to decipher the impact of training on one day and validate it on another one. The impact of seasonal fluctuations in clear sky irradiance was examined by validating the spring equinox-trained algorithms on 43,000 degradation paths generated from clear sky irradiance on the first day of each month for a year, Fig. 2a. This was done twice with two distinct sets of cell parameters (details are in Supplementary Table 1, cells 5–28) to investigate the impact of cell-to-cell variations at the same time. This impact will be assessed by comparing the diagnosis statistics for the two different batches of cells comprising each 12 × 43,000 data points. The impact of irradiance variations was significant as the average RMSE increased by 1.6% for *Q*-diagnosis and by more than 2% for the *t*-diagnosis compared to the ideal scenario, Table 1 bottom 4 rows. The three NN methods were the best performing for *Q*-diagnosis, with RMSE below 1% for 25% or less degradation and below 2% for 50% or less degradation. For the *t*-diagnosis, all the algorithms but DTW-CNN performed similarly, with RMSE slightly over 3% for 25% or less degradation (around 5% for 50% or less degradation). LAM_NE_ diagnosis still had the highest RMSE, with LLI and LAM_PE_ RMSEs being close. LAM_PE_ RMSEs were lower for *Q*-diagnosis and the LLI ones for *t*-diagnosis. Cell-to-cell variations were negligible, with, on average, 0.4% MAE with a 0.7% standard deviation between the two sets, Supplementary Fig. 2f.

The third set of experiments examined the impact of different cloud coverages. This corresponds to the validation using observations. To remove the effects of a time difference between training and validation data, training and validation data corresponded in time. For each of the 18 cloudy days detailed in the “Irradiance data selection” section, validation of algorithms trained on clear sky irradiance was performed using irradiance observations for that day, which included cloud effects. Table 2 presents the average statistics for all 18 days, for days with at least 50% clear skies (10 out of 18 days), and for days with at least 75% clear skies (3 out of 18 days). Complete statistics with MAE and *ρ* are available in Supplementary Tables 3 and 4. Overall, for degradation paths with less than 25% of each degradation mode, the RMSEs were in the 1.75–3.6% range for all algorithms for *Q*-diagnosis and in the 4.4–5.2% range for *t*-diagnosis. Focusing on clearer days reduced the RMSE significantly to below 1% (FNN, 1DConv, DTW-CNN) for *Q*-diagnosis and 2.5% (XGB, RF, 1DConv) for *t*-diagnosis. This highlights the validity of diagnosis under real irradiance conditions with cloud effects.

**Table 2:** RMSE summary statistics for diagnosis on 11,000 voltage vs. capacity curves generated for degradation below 25% for all days, days with 50% of more clear sky, and days with 75% or more clear sky

	RF	XGB	FNN	1DConv	DTW-CNN
*Q*-based, all days, <25% deg.	3.55	3.25	2.30	2.33	1.76
*Q*-based, all days, <50% deg.	4.89	4.84	3.92	4.19	2.91
*t*-based, all days, <25% deg.	4.46	5.01	5.20	4.70	5.17
*t*-based, all days, <50% deg.	5.84	6.25	8.53	8.59	7.67
*Q*-based, >50% clear sky, <25% deg.	1.91	1.75	1.15	1.02	0.78
*Q*-based, >50% clear sky, <50% deg.	2.91	3.04	2.02	2.04	1.67
*t*-based, >50% clear sky, <25% deg.	3.01	3.61	4.34	3.59	4.20
*t*-based, >50% clear sky, <50% deg.	4.67	4.99	7.80	7.14	6.67
*Q*-based, >75% clear sky, <25% deg.	1.37	1.22	0.85	0.72	0.58
Q-based, >75% clear sky, <50% deg.	2.41	2.56	1.59	1.65	1.16
*t*-based, >75% clear sky, <25% deg.	2.45	2.23	4.15	2.45	3.06
*t*-based, >75% clear sky, <50% deg.	3.14	4.07	7.43	5.03	4.78

Algorithms were trained on the same day to clear sky irradiance.

Associated full statistics are available in Supplementary Tables 3 and 4.

## Discussion

**Fig. 7:**
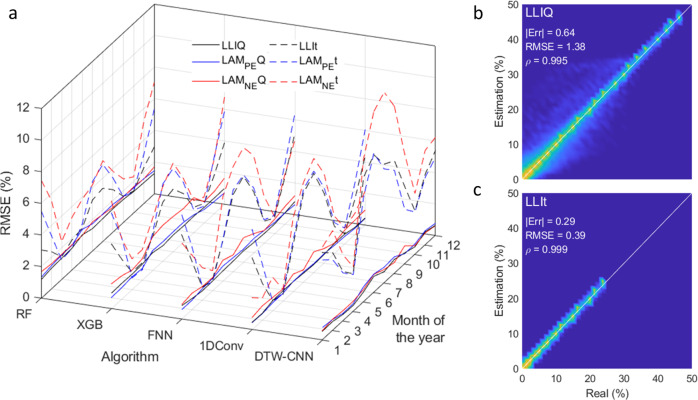
RMSE dependency on the day of the year and predicted vs. real LLI estimation distribution as a function of degradation extent. **a** RMSE dependency of the day of the year for all five algorithms trained on data from the spring equinox (black: LLI, blue: LAM_PE_, red: LAM_NE_, full lines: *Q*-based diagnosis, dashed lines: *t*-based diagnosis). **b** Predicted vs. real LLI distribution for the DTW-CNN algorithm when trained and validated on the same day for the entire dataset with up to 50% degradation for each degradation mode. **c** Predicted vs. real LLI distribution for the DTW-CNN algorithm when trained and validated on the same day for the entire dataset with up to 25% degradation for each degradation mode.

This study provides the application of synthetic datasets for non-CC simulations. Because the current was not constant, capacity and time were uncorrelated, which offered an opportunity to study two different datasets, *V* vs. *Q* and *V* vs. *t*. While using voltage versus capacity is standard, it might not be the best solution for deployed systems because the *V* vs. *t* dataset should be less error-prone than the V vs. *Q* one, as capacity is not directly measurable but derived from time and current^46^. The *V* vs. *Q* dataset is, however, expected to be easier to diagnose because the area under a d*Q*/d*V* peak corresponds to capacity, and, at low rates, it is independent of the applied current because it has a finite value. Therefore, current variations should have a limited impact on the overall peak shape and intensity. This is why the voltage responses showcased in Fig. 6a–c are really similar to the signature under CC^21^ despite the current varying. This is not the case for d*t*/d*V* peaks because, while capacity stays the same with varying currents, the time taken to complete the peak will be different. Therefore d*t*/d*V* peaks are much more sensitive to changes in current than d*Q*/d*V* ones. This sensibility explains the differences observed between Fig. 6d–f and Fig. 6a–c and why the *t*-diagnoses average errors were, on average more than double the *Q*-based ones. The increased error was especially visible when the validation was done on a duty cycle different from the one used for training. Figure 7a plots the RMSE variations as a function of the month of the year for algorithms trained on one day only (second set of experiments). The *Q*-based RMSE showcased little to no effect of the month of the year, whereas the *t*-based ones varied significantly with a minimum close to the training day (March and April) and in fall (September and October) when irradiances are the most similar to the one used for training (spring equinox), Fig. 2b. The difference was also much more pronounced for cloudy days and aged cells. Therefore, while *t*-diagnosis is more interesting on paper, it might not be the best solution where clear sky did not significantly dominate, at least for the tested algorithms.

Supplementary Figure 2, as well as Tables 1 and 2, showed the performance degradation with increasing degradation percentage. This decline in performance can be explained by multiple factors. Although data imbalance during training could be a possible factor, as 2/3rds of the training data has a degradation below 25%, the main factor seems to come from the fact that small variations in one of the three degradation modes are hard to quantify when at least one of the other two modes has large variations. This is exemplified in Fig. 7b, c, where a distribution of the estimated vs. true values for LLI and the DTW-CNN algorithms are plotted for 50% or less and 25% or less degradation. For the 50% or less degradation, there is a haze around the 1:1 line below 20% LLI that disappears when the maximum degradation is set at 25%. This indicates that the error mostly comes from degradation paths with low LLI but at least one LAM above 25%.

Looking at the detailed statistics, it can be seen from Table 2 as well as Supplementary Tables 3 and 4 that although the algorithms’ performance was close, some differences were noticeable. Overall, the DTW-CNN algorithm offers the best performance for *Q*-diagnosis, while 1DConv is better for *t*-diagnosis for degradations below 25%. Moreover, the algorithms are not all affected the same by the change of duty cycles. This is especially visible in Fig. 7a, where the performance of the *t*-diagnosis was much more affected for winter days than for summer days for RF, while the opposite is true for DTW-CNN; the other three algorithms were impacted the same. Looking in more detail, it appears that the largest errors were always observed for LAM_NE_ estimation (Supplementary Tables 2–4). This could be explained by the fact that LAM_NE_ cannot be directly inferred from any feature of interest of the IC or IT curves. For the other two degradation modes, and as showcased in previous work^21^, the intensity of high voltage shoulder is in most cases directly proportional to LAM_PE_ and the intensity of the main peak to LLI. This is specific to the nickel manganese cobalt oxide positive electrode, and different results are expected for non-layered oxides such as LiBs with LiFePO_4_-based positive electrodes where LAM_PE_ should be much harder to quantify than the other two. A possible solution to improve the accuracy of LAM_NE_ estimation for the current algorithms could be to train the algorithms on dV/dQ vs. Q curves on top of the IC curves, as LAM_NE_ is, in most cases, directly decipherable from these.

**Fig. 8:**
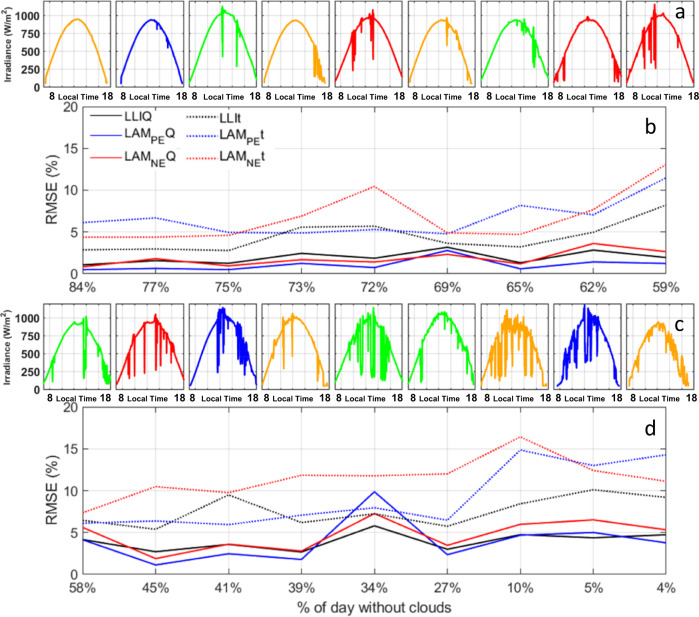
RMSE dependency of clear sky percentage and cloud distribution. Evolution of RMSE for the DTW-CNN algorithm as a function of the clear sky percentage (black: LLI, blue: LAM_PE_, red: LAM_NE_, full lines: *Q*-based diagnosis, dotted lines: *t*-based diagnosis) for the 18 days considered in this work sorted from lowest to highest clear sky coverage, see Fig. 3c. **a** Irradiances and **b** average RMSEs for days with 59% or more clear sky. **c** Irradiances and **d** average RMSEs for days with less than 59% clear sky. The irradiance data is color coded to showcase seasonality (spring in green, summer in red, fall in orange, and winter in blue, see Fig. 3b).

Figure 8 presents the RMSE variation for all the cloudy days tested in this work sorted by their clear sky percentage with the associated actual irradiance vs. time curve as inset. Overall, clear sky percentage is a useful indicator of diagnosability, although other parameters also come into play. In general, the RMSE increases as the clear sky percentage decreases. However, there were some duty cycles that showed abnormal high (e.g., 34% clearness and 59% clearness) or low (e.g., 27% clearness) RMSE, indicating that the intensity and time of the cloud coverage could also play a key role in diagnosability. Together with cloud coverage, the type of diagnosis, *Q*-based or *t*-based also has a role. For example, for a day with a 59% clear sky, *Q*-diagnosis was better than normal and the *t*-based one far worse, while for a day with a 34% clear sky, the opposite was true. Finding the right set of parameters to identify which days are more auspicious for the diagnosis will require more work, but from these results, it is clear that the use of synthetic data will be instrumental in evaluating the impact of different classification schemes.

In summary, we propose a data-driven approach for the diagnosis of LiBs paired with PV using synthetic data. This approach allows the degradation of PV-connected LiBs to be diagnosed without the need for maintenance cycles by using state-of-art ML algorithms. Diagnoses were obtained with an average RMSE of 2.75% for more than 10,000 different degradation paths with 25% or less of the three thermodynamic degradation modes. Because the diagnosis was made outside of CC, the capacity- and time-based information could be decorrelated and compared. The time-based diagnosis was shown to be less accurate than its capacity counterpart for the tested algorithms. However, the accuracy of both types of diagnosis is satisfactory for days when clear sky dominates. For days with lower clear sky conditions, accuracy depends on the clear sky percentage, but additional factors such as the time and duration of cloud coverage also come into play.

The framework presented here proved that opportunistic diagnosis of LiBs connected to PV is possible from auspicious cloud coverages. Based on our results and for the studied system and location, the diagnosis could be possible one out of every five days independent of the season, which is more than frequent enough for LiBs supposed to last 3500 days or more. This number might be different in other locations where shadowing or snow could play a significant role, but it could be assessed using adapted synthetic datasets.

Despite promising results, there is still a significant amount of work to be done before this technique can be applied to deployed systems. There is a need for training under a wide array of different conditions, as PV systems in the field will have varying orientations, tilt, locations, cleanliness, etc. Moreover, this work was performed on single cells and without considering any additional usage of the cells. Real systems will be composed of battery packs, which will have varying voltage responses due to inhomogeneities and imbalance. Furthermore, these batteries will likely be used at the same time they are charged, which will further modify the duty cycles. The validation framework provided here can be applied to study these case figures, and future work will address the impact of geographic locations, module size, and additional loads on the LiBs. The proposed framework might even apply to other types of intermittent renewable power systems for which storage could be considered, such as wave or tidal energy.

## Methods

### PV data acquisition

The PV testbed used in this work includes instrumentation for high-frequency PV and solar resource monitoring, including a Kipp & Zonen SMP21-A secondary standard pyranometer, which is installed in the POA of the testbed PV panels. The data was collected at 1 s intervals and averaged to 1 min for 2 years.

### Battery testing

The commercial cells used in this work were provided by an industrial partner and are composed of a graphite-based NE and a nickel manganese cobalt oxide positive electrode (PE) with a 1:1:1 stoichiometry. The industrial partner also provided the full cell cycling data with C/15, C/8.5, and C/4 cycles performed on a pristine cell. The electrode materials for the assembly of lab-scale Li metal cells were harvested and sampled after the disassembly of a commercial cell from the same batch. The commercial Li-ion cell was discharged to 2.0 V at C/50 before being opened in an Argon-filled glove box [<0.1 ppm O_2_ and H_2_O]. The double-side coated electrodes were rinsed with dimethyl carbonate [2cl, 99% anhydrous, Sigma Aldrich], and one side was scrubbed using N-Methylpyrrolidone [Biotech grade solvent, 99.5+%, Sigma Aldrich] before 1.8 mm diameter electrodes were cut using an EL-CUT punching tool (EL-CELL, Hamburg, Germany). Lab-scale Li metal cells were assembled in PAT-CELLs (EL-CELL, Hamburg, Germany) using a standard polypropylene sleeve, a borosilicate glass fiber separator [Whatman GF/A, 0.26 mm thickness, 1.6 µm pores], a metallic Li NE [99.9% trace metal basis, 0.38 mm thickness, Sigma Aldrich], as well as 300 µl of an electrolyte composed of ethylene carbonate [≥99%, acid <10 ppm, H_2_O < 10 ppm, Sigma Aldrich] and propylene carbonate [anhydrous, 99.7%, Sigma Aldrich] in a 1:1 weight ratio with 1 M Lithium hexafluorophosphate [>99.99% trace metals basis, Sigma Aldrich] and 2% weight vinylene carbonate [99.5%, acid <200 ppm, H_2_O < 100 ppm, Sigma Aldrich]. For the testing, the cell formation consisted of 8 cycles at C/10 followed by 1 cycle at C/25 between 3.2 V and 4.3 V for the PE and 0.02 and 1.2 V for the NE. After the formation cycles, the cells were tested at C/50, C/25, C/15, C/8, C/4, C/2, and C/1 with residual capacity measurements at C/50 for each regime with 4-hour rests before and after^47^. The test was repeated three times for reproducibility.

### Synthetic data generation

The synthetic data used in this work, both for training and validation, was generated by scanning the entire range of possible combinations for LLI and LAMs^21–23^. Because the duty cycles have maximum currents below C/6, only the thermodynamic degradation modes were considered in this work. The maximum value for the degradation modes was set at 50%. For the main training dataset, the composition resolution was set at 1% (5000 [LLI, LAM_PE_, LAM_NE_] triplet tested) with at most a simulation every 0.5% for each degradation mode (>125 simulations per triplet from 0 to 50%). This resulted in around 700,000 unique voltage responses for training. Additional training on different duty cycles was done with a 2.5% resolution with 1% steps to limit file sizes. This corresponds to more than 850 different triplets and 43,000 unique voltage curves. For the validation datasets, the resolution was decreased to 5% (225 triplets) with 1% steps (50 simulations per triplet), resulting in around 11,000 curves per condition.

Finally, to avoid any overfitting error by training and validating on the same data, each simulation will be performed on a slightly different cell, i.e., a cell with emulation parameters (LR, OFS, R, and RDFs) randomly varied by ±1% to be in the same range as observed cell-to-cell variations in commercial cells^48^. The overall parameters for each simulation with the associated duty cycles are summarized in Supplementary Table 1.

### Diagnosis algorithms

In this work, the leading ML algorithms for degradation modes quantification were used to validate our approach. A thermodynamic degradation modes diagnosis corresponds to the quantification of LLI and LAMs for the PE and NE, respectively^43,49^. Such quantification provides more information than a simple capacity estimation and enables prognosis^21^. The selected algorithms can be divided into two categories, decision tree ensemble methods, and neural networks. Decision trees are deterministic models that rely on multiple conditionals, while neural networks follow a probabilistic approach in which they seek to learn by activating artificial neurons. For this work, RF^25^ and XGB^26^ algorithms were selected for the decision trees, and FNN^27^, 1D-CNN (1DConv)^20^, and the DTW-CNN approach^28^ were selected as neural networks. It is important to note that in all cases, the models use the raw derivative voltage curves as input except for DTW-CNN, which uses images created from the DTW matrix between the pristine and the degraded derivative curves. This allows to transformation of voltage changes into images that reflect the degradation and enables the use of 2D CNNs, which are widely known in the literature to work well with images^28^.

In terms of implementation and for the decision tree ensemble methods:

- The sklearn library^50^ was used to implement the Random Forrest, specifically the ensemble module with the RandomForestRegressor algorithm, the hyperparameters were max_depth, and n_estimators.
- For the XGBoost model, the xgboost library^51^ was used, specifically the XGBRegressor algorithm, the hyperparameters were max_depth and eta.

For the neural networks, all the models were implemented in TensorFlow^52^. The models’ configurations were as follows:

- FFN: 3 fully connected layers with 64 neurons in the first layer, 32 in the second, and 3 in the third.
- CNN-1D: 5 layers, of which 2 are CNN-1D layers with 32 neurons each, and 3 are fully connected layers with 128, 64 and 3 neurons each.
- CNN-DTW: 4 convolution layers with 64 neurons in the first two layers and 128 in the next two, followed by two fully connected layers with 512 and 3 neurons each.

The hyperparameters to be set in these three cases were the batch size and learning rate.

The WandB framework^53^ was used for hyperparameter tuning, and callbacks were used during training to relegate the training stop condition to the validation error instead of the number of epochs.

In this work, validation comprised varying initial conditions for the ML algorithms to produce model output that is compared against some truth to generate error statistics, which are used to quantify the experiments.

Further details regarding the experimental setup and the source code to reproduce the experimental results are available in a public git repository^54^.

### Statistical testing

Formulas for statistical tests:

1 $${{{{{\rm{RMSE}}}}}}=\sqrt{\mathop{\sum }\limits_{i=1}^{n}\frac{{\left({y}_{i}-{x}_{i}\right)}^{2}}{n}.}$$

2 $${{{{{\rm{MAE}}}}}}=\frac{{\sum }_{i=1}^{n}\left|{y}_{i}-{x}_{i}\right|}{n}.$$

3 $$\rho=\,\frac{{\sum }_{i=1}^{n}({x}_{i}-\bar{x})({y}_{i}-\bar{y})}{\sqrt{{\sum }_{i=1}^{n}{\left({x}_{i}-\bar{x}\right)}^{2}\mathop{\sum }\nolimits_{i=1}^{n}{\left({y}_{i}-\bar{y}\right)}^{2}}}.$$

With *y*_*i*_ the prediction, $\bar{{y}}$ the prediction mean, ${{x}}_{{i}}$ the true value, $\bar{{x}}$ the true mean, and ${n}$ the total number of data points.

### Reporting summary

Further information on research design is available in the Nature Portfolio Reporting Summary linked to this article.

## Supplementary information

Supplementary Information

Reporting Summary

Solar Cells Reporting Summary

## Data Availability

The sharable data generated in this study have been deposited in a Mendeley database^30,31^ that contains the photovoltaics data as well as the synthetic cycles for perfect irradiance and cloud coverage.

## Code availability

The source code to reproduce the experimental results associated with the ML algorithms is available at https://github.com/NahuelCostaCortez/PVDiagnosis^54^.
