# Supplementary material for: Data-driven direct diagnosis of Li-ion batteries connected to photovoltaics
Source: Nat Commun. 2023 May 30;14:3138. doi: 10.1038/s41467-023-38895-7 (PMC10229535; doi:10.1038/s41467-023-38895-7)
Supplement: Supplementary file 1 — Supplementary Information [file 41467_2023_38895_MOESM1_ESM.pdf]

Supplementary Information

Data-driven direct diagnosis of Li-ion batteries connected to photovoltaics

Matthieu Dubarry[a]*, Nahuel Costa[b], and Dax Matthews[a]

[a]Hawai‘i Natural Energy Institute, University of Hawai‘i at Mānoa, 1680 East West Road, POST 109, Honolulu, HI, 96822 USA

[b]Computer Science Department, Polytechnic School of Engineering, University of Oviedo, Gijon, 33202, Asturias, Spain

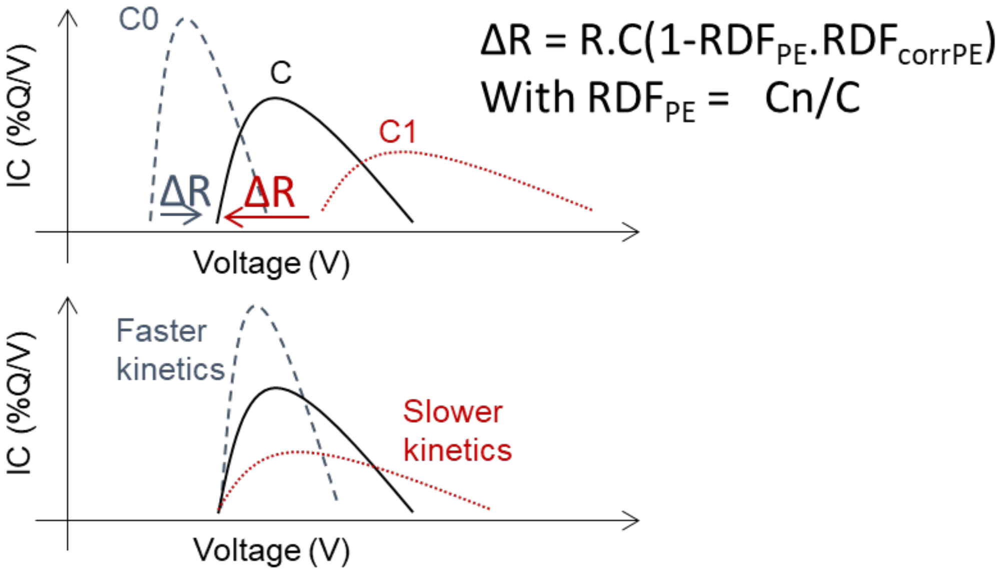

Supplementary Figure 1. Kinetic accommodation Principles. R is the cell resistance, C the requested rate, RDF_{PE} the rate degradation factor, and RDF_{corrPE} the additional correction needed.

Supplementary Figure 2. RMSE dependency on maximum degradation of at least one of the degradation modes and impact of cell-to-cell variations. a-e RMSE dependency for the RF, XGB, FNN, 1D-Conv, and DTW-CNN respectively. **f** diagnosis error induced by cell-to-cell variations for more than 14,000,000 samples.

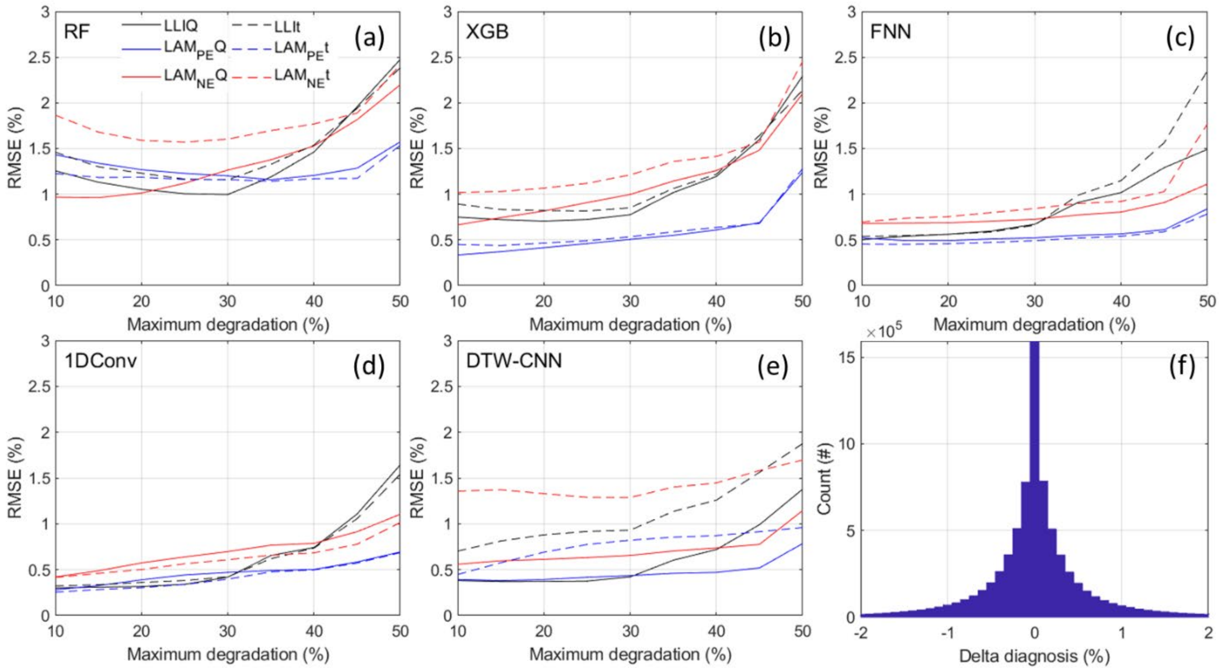

Supplementary Table 1. Summary of all simulations performed in this work with emulation parameters (PI: perfect irradiance, C: cloudy).

Cell #	LR	OFS	R	RDF_{PE} a	RDF_{PE} b	RDF_{CorrPE} a	RDF_{CorrPE} b	RDF_{NE} a	RDF_{NE} b	Duty cycle	Paths Resolution
1	1.200	4.000	-0.100	0.064	-0.836	3.916	-0.306	0.443	-0.217	PI - 03/21/2017	1%
2	1.199	4.033	-0.099	0.064	-0.840	3.921	-0.304	0.444	-0.216	PI - 03/21/2017	1%
5	1.195	3.964	-0.100	0.064	-0.838	3.884	-0.303	0.445	-0.219	PI - 01/01/2017	2.5%
6	1.201	3.969	-0.101	0.064	-0.833	3.935	-0.303	0.439	-0.218	PI - 02/01/2017	2.5%
7	1.202	4.002	-0.100	0.064	-0.841	3.899	-0.307	0.444	-0.217	PI - 03/01/2017	2.5%
8	1.189	4.022	-0.100	0.064	-0.840	3.885	-0.304	0.443	-0.217	PI - 04/01/2017	2.5%
9	1.209	4.024	-0.100	0.063	-0.829	3.884	-0.308	0.447	-0.218	PI - 05/01/2017	2.5%
10	1.191	4.018	-0.099	0.064	-0.838	3.903	-0.307	0.445	-0.217	PI - 06/01/2017	2.5%
11	1.206	3.979	-0.100	0.065	-0.842	3.884	-0.305	0.442	-0.218	PI - 07/01/2017	2.5%
12	1.202	4.023	-0.100	0.064	-0.829	3.937	-0.304	0.442	-0.217	PI - 08/01/2017	2.5%
13	1.193	4.011	-0.100	0.064	-0.841	3.885	-0.305	0.441	-0.217	PI - 09/01/2017	2.5%
14	1.190	3.992	-0.099	0.064	-0.841	3.900	-0.307	0.447	-0.217	PI - 10/01/2017	2.5%
15	1.205	4.021	-0.100	0.064	-0.829	3.950	-0.304	0.441	-0.218	PI - 11/01/2017	2.5%
16	1.200	4.022	-0.100	0.064	-0.828	3.930	-0.306	0.443	-0.217	PI - 12/01/2017	2.5%
17	1.189	3.985	-0.101	0.064	-0.830	3.887	-0.304	0.439	-0.217	PI - 01/01/2017	2.5%
18	1.204	4.018	-0.100	0.063	-0.838	3.887	-0.304	0.439	-0.215	PI - 02/01/2017	2.5%
19	1.192	3.976	-0.100	0.064	-0.831	3.897	-0.308	0.445	-0.217	PI - 03/01/2017	2.5%
20	1.192	3.977	-0.099	0.065	-0.839	3.921	-0.305	0.440	-0.218	PI - 04/01/2017	2.5%
21	1.212	3.974	-0.100	0.064	-0.829	3.930	-0.305	0.447	-0.217	PI - 05/01/2017	2.5%
22	1.203	3.972	-0.100	0.064	-0.840	3.945	-0.305	0.445	-0.216	PI - 06/01/2017	2.5%
23	1.201	4.027	-0.100	0.064	-0.833	3.912	-0.306	0.442	-0.217	PI - 07/01/2017	2.5%
24	1.206	3.994	-0.100	0.064	-0.828	3.900	-0.305	0.444	-0.219	PI - 08/01/2017	2.5%
25	1.210	3.997	-0.099	0.064	-0.840	3.935	-0.307	0.440	-0.218	PI - 09/01/2017	2.5%
26	1.199	3.977	-0.099	0.064	-0.831	3.890	-0.307	0.446	-0.217	PI - 10/01/2017	2.5%
27	1.205	3.972	-0.101	0.064	-0.839	3.880	-0.308	0.445	-0.215	PI - 11/01/2017	2.5%
28	1.201	3.986	-0.100	0.064	-0.835	3.891	-0.305	0.439	-0.219	PI - 12/01/2017	2.5%
29	1.204	4.035	-0.099	0.065	-0.841	3.922	-0.306	0.441	-0.218	C - 01/05/2017	5%
30	1.193	3.965	-0.101	0.064	-0.840	3.927	-0.306	0.442	-0.218	C - 11/11/2016	5%
31	1.196	4.025	-0.101	0.064	-0.836	3.927	-0.309	0.443	-0.215	C - 11/14/2016	5%
32	1.209	4.010	-0.100	0.065	-0.831	3.928	-0.307	0.442	-0.215	C - 11/16/2016	5%
33	1.189	3.994	-0.099	0.064	-0.834	3.943	-0.307	0.444	-0.216	C - 04/17/2016	5%
34	1.211	3.981	-0.101	0.064	-0.834	3.884	-0.307	0.440	-0.215	C - 07/28/2016	5%
35	1.205	3.988	-0.100	0.064	-0.838	3.879	-0.309	0.446	-0.218	C - 06/13/2016	5%
36	1.208	3.991	-0.100	0.064	-0.837	3.898	-0.304	0.443	-0.216	C - 11/23/2016	5%
37	1.207	4.039	-0.099	0.064	-0.829	3.940	-0.309	0.439	-0.219	C - 07/26/2016	5%
38	1.188	4.015	-0.101	0.064	-0.842	3.947	-0.307	0.440	-0.216	PI - 11/11/2016	2.5%
39	1.192	3.963	-0.099	0.064	-0.843	3.905	-0.305	0.447	-0.219	PI - 11/14/2016	2.5%

**40**	1.204	4.039	-0.101	0.064	-0.839	3.896	-0.305	0.445	-0.217	PI - 01/05/2017	2.5%
**41**	1.198	4.008	-0.101	0.064	-0.837	3.923	-0.306	0.439	-0.218	PI - 04/17/2016	2.5%
**42**	1.212	3.988	-0.101	0.064	-0.842	3.912	-0.305	0.440	-0.215	PI - 07/28/2016	2.5%
**43**	1.195	4.018	-0.101	0.064	-0.828	3.943	-0.309	0.445	-0.215	PI - 11/16/2016	2.5%
**44**	1.197	4.016	-0.100	0.064	-0.832	3.930	-0.306	0.444	-0.216	PI - 06/13/2016	2.5%
**45**	1.192	4.026	-0.101	0.065	-0.829	3.891	-0.304	0.443	-0.216	PI - 07/27/2016	2.5%
**46**	1.210	3.968	-0.099	0.064	-0.841	3.901	-0.304	0.442	-0.216	PI - 11/23/2016	2.5%
**47**	1.200	4.033	-0.100	0.063	-0.834	3.881	-0.306	0.442	-0.219	PI - 03/31/2017	2.5%
**48**	1.207	3.999	-0.101	0.064	-0.834	3.949	-0.309	0.445	-0.218	PI - 06/01/2017	2.5%
**49**	1.196	4.035	-0.099	0.064	-0.838	3.942	-0.305	0.445	-0.218	PI - 10/19/2017	2.5%
**50**	1.196	4.004	-0.101	0.064	-0.833	3.925	-0.305	0.445	-0.217	PI - 03/11/2017	2.5%
**51**	1.191	4.033	-0.099	0.064	-0.843	3.916	-0.307	0.444	-0.218	PI - 08/02/2017	2.5%
**52**	1.189	4.002	-0.099	0.064	-0.833	3.943	-0.304	0.444	-0.219	PI - 09/18/2017	2.5%
**53**	1.189	3.964	-0.099	0.064	-0.838	3.886	-0.308	0.444	-0.215	PI - 02/01/2017	2.5%
**54**	1.190	3.971	-0.101	0.063	-0.832	3.896	-0.304	0.446	-0.218	PI - 05/22/2017	2.5%
**55**	1.206	4.012	-0.100	0.064	-0.839	3.886	-0.304	0.439	-0.219	PI - 10/17/2017	2.5%
**56**	1.211	3.970	-0.100	0.064	-0.832	3.936	-0.306	0.442	-0.216	C - 03/31/2017	5%
**57**	1.206	4.032	-0.100	0.064	-0.843	3.897	-0.306	0.447	-0.216	C - 06/01/2017	5%
**58**	1.194	4.034	-0.099	0.064	-0.838	3.893	-0.307	0.446	-0.217	C - 10/19/2017	5%
**59**	1.204	4.024	-0.099	0.064	-0.830	3.917	-0.308	0.447	-0.217	C - 03/11/2017	5%
**60**	1.195	4.012	-0.101	0.064	-0.844	3.892	-0.304	0.441	-0.217	C - 08/02/2017	5%
**61**	1.198	3.985	-0.100	0.063	-0.834	3.900	-0.305	0.440	-0.217	C - 09/18/2017	5%
**62**	1.195	3.972	-0.099	0.064	-0.837	3.945	-0.303	0.447	-0.215	C - 02/01/2017	5%
**63**	1.208	4.024	-0.101	0.064	-0.836	3.909	-0.304	0.444	-0.217	C - 05/22/2017	5%
**64**	1.193	4.002	-0.101	0.064	-0.839	3.909	-0.303	0.441	-0.218	C - 10/17/2017	5%

Supplementary Table 2: Complete statistics for the validation on the same day (sample size 100,000 points) and on different days (sample size 43,000 points) for the full dataset and degradations limited to 25% for each degradation mode.

<25% degradation	RF			XGB			FNN			1DConv			DTW-CNN		
	RMSE (%)	MAE (%)	ρ	RMSE (%)	MAE (%)	ρ	RMSE (%)	MAE (%)	ρ	RMSE (%)	MAE (%)	ρ	RMSE (%)	MAE (%)	ρ
LLI(Q), same day	0.97	0.61	0.992	0.71	0.48	0.995	0.62	0.50	0.997	0.37	0.28	0.999	0.39	0.29	0.999
LAMPE(Q), same day	1.18	0.72	0.988	0.46	0.31	0.998	0.52	0.40	0.998	0.46	0.34	0.998	0.43	0.33	0.999
LAMNE(Q), same day	1.12	0.81	0.989	0.92	0.68	0.993	0.72	0.54	0.996	0.67	0.50	0.996	0.64	0.49	0.997
Mean, same day	1.09	0.71	0.990	0.70	0.49	0.996	0.62	0.48	0.997	0.50	0.37	0.998	0.49	0.37	0.998
LLI(t), same day	1.13	0.75	0.990	0.80	0.55	0.994	0.61	0.49	0.997	0.40	0.31	0.999	0.92	0.74	0.998
LAMPE(t), same day	1.12	0.71	0.989	0.49	0.34	0.998	0.48	0.39	0.998	0.37	0.28	0.999	0.81	0.63	0.997
LAMNE(t), same day	1.55	1.07	0.977	1.15	0.86	0.988	0.82	0.65	0.995	0.59	0.44	0.997	1.28	1.02	0.992
Mean, same day	1.27	0.85	0.986	0.81	0.58	0.993	0.64	0.51	0.996	0.45	0.34	0.998	1.00	0.80	0.996
LLI(Q), different day	1.38	1.03	0.987	1.02	0.75	0.991	0.68	0.53	0.996	0.55	0.45	0.998	0.68	0.57	0.998
LAMPE(Q), different day	1.61	1.22	0.983	0.97	0.72	0.995	0.76	0.64	0.997	0.51	0.40	0.998	0.84	0.72	0.997
LAMNE(Q), different day	1.59	1.22	0.978	1.73	1.09	0.970	1.08	0.82	0.991	0.91	0.71	0.995	0.84	0.66	0.995
Mean, different day	1.53	1.16	0.982	1.24	0.85	0.985	0.84	0.66	0.995	0.66	0.52	0.997	0.79	0.65	0.997
LLI(t), different day	2.35	1.73	0.950	2.58	2.07	0.962	3.09	2.49	0.966	3.02	2.46	0.960	5.36	4.60	0.928
LAMPE(t), different day	3.14	2.47	0.923	3.28	2.86	0.967	3.50	2.83	0.955	3.63	2.90	0.947	4.89	3.94	0.909
LAMNE(t), different day	4.41	2.83	0.807	3.85	2.85	0.828	4.10	3.00	0.888	3.10	2.38	0.947	7.01	4.99	0.630
Mean, different day	3.30	2.34	0.893	3.24	2.59	0.919	3.56	2.77	0.937	3.25	2.58	0.951	5.75	4.51	0.822
<50% degradation	RF			XGB			FNN			1DConv			DTW-CNN		
	RMSE (%)	MAE (%)	ρ	RMSE (%)	MAE (%)	ρ	RMSE (%)	MAE (%)	ρ	RMSE (%)	MAE (%)	ρ	RMSE (%)	MAE (%)	ρ
LLI(Q), same day	2.48	1.24	0.983	2.29	1.10	0.985	1.49	0.88	0.994	1.64	0.74	0.993	1.38	0.64	0.995
LAMPE(Q), same day	1.57	0.83	0.993	1.24	0.54	0.996	0.84	0.51	0.998	0.69	0.45	0.999	0.79	0.45	0.998
LAMNE(Q), same day	2.20	1.30	0.987	2.10	1.15	0.989	1.11	0.73	0.997	1.10	0.73	0.997	1.14	0.64	0.996
Mean, same day	2.08	1.12	0.988	1.88	0.93	0.990	1.15	0.71	0.996	1.15	0.64	0.996	1.10	0.58	0.997
LLI(t), same day	2.38	1.30	0.985	2.14	1.13	0.987	2.35	1.07	0.984	1.54	0.72	0.994	1.88	1.08	0.991
LAMPE(t), same day	1.54	0.87	0.994	1.28	0.56	0.996	0.79	0.49	0.998	0.69	0.48	0.999	0.96	0.75	0.998
LAMNE(t), same day	2.42	1.50	0.985	2.45	1.34	0.984	1.77	0.89	0.991	1.01	0.64	0.997	1.70	1.25	0.993
Mean, same day	2.11	1.22	0.988	1.96	1.01	0.989	1.64	0.82	0.991	1.08	0.61	0.997	1.51	1.03	0.994
LLI(Q), different day	3.23	1.79	0.973	3.00	1.58	0.976	1.95	1.03	0.990	2.03	0.97	0.990	2.02	1.05	0.989
LAMPE(Q), different day	2.23	1.31	0.988	2.10	1.05	0.990	1.39	0.80	0.996	1.23	0.57	0.996	1.13	0.83	0.997
LAMNE(Q), different day	2.95	1.86	0.978	4.34	2.15	0.944	1.80	1.12	0.992	1.93	1.01	0.991	1.43	0.96	0.995
Mean, different day	2.80	1.65	0.980	3.15	1.59	0.970	1.71	0.98	0.993	1.73	0.85	0.992	1.53	0.95	0.994
LLI(t), different day	3.69	2.55	0.967	3.97	2.85	0.969	4.99	3.29	0.954	4.25	3.05	0.968	7.50	6.03	0.890
LAMPE(t), different day	4.38	3.12	0.964	4.21	3.32	0.979	4.11	3.26	0.982	4.59	3.63	0.978	5.59	4.52	0.923
LAMNE(t), different day	5.21	3.61	0.929	5.09	3.58	0.934	7.21	4.66	0.902	4.51	3.26	0.962	9.76	7.36	0.727
Mean, different day	4.43	3.10	0.953	4.42	3.25	0.961	5.43	3.74	0.946	4.45	3.31	0.969	7.62	5.97	0.847

Supplementary Table 3: Complete statistics for the validation on cloudy days when less than 25% of each degradation modes (sample size 11,000 points).

<25% degradation	RF			XGB			FNN			1DConv			DTW-CNN		
	RMSE (%)	MAE (%)	ρ	RMSE (%)	MAE (%)	ρ	RMSE (%)	MAE (%)	ρ	RMSE (%)	MAE (%)	ρ	RMSE (%)	MAE (%)	ρ
LLI(Q), all days	3.03	2.17	0.868	2.96	2.05	0.855	2.07	1.65	0.917	2.11	1.56	0.923	1.40	1.13	0.980
LAM_{PE}(Q), all days	3.33	2.35	0.813	2.71	1.82	0.904	2.18	1.70	0.912	2.58	1.83	0.896	1.79	1.44	0.969
LAM_{NE}(Q), all days	4.29	3.03	0.801	4.07	3.07	0.825	2.65	2.04	0.887	2.31	1.65	0.919	2.09	1.59	0.959
Mean, all days	3.55	2.52	0.827	3.25	2.31	0.862	2.30	1.80	0.905	2.33	1.68	0.913	1.76	1.39	0.969
LLI(t), all days	3.47	2.61	0.895	3.35	2.32	0.888	3.74	2.78	0.916	3.37	2.48	0.903	4.04	3.10	0.909
LAM_{PE}(t), all days	3.82	2.88	0.868	4.33	3.06	0.910	4.14	3.14	0.904	5.04	3.49	0.789	4.70	3.45	0.858
LAM_{NE}(t), all days	6.07	4.47	0.747	7.35	6.09	0.771	7.71	5.02	0.715	5.70	4.16	0.738	6.77	5.46	0.630
Mean, all days	4.46	3.32	0.837	5.01	3.82	0.856	5.20	3.64	0.845	4.70	3.38	0.810	5.17	4.01	0.799
LLI(Q), >50% clear sky	1.51	1.07	0.975	1.24	0.81	0.977	0.98	0.79	0.989	0.88	0.66	0.995	0.62	0.49	0.997
LAM_{PE}(Q), >50% clear sky	1.60	1.10	0.966	1.51	0.91	0.974	1.06	0.84	0.989	1.04	0.75	0.991	0.73	0.57	0.995
LAM_{NE}(Q), >50% clear sky	2.63	1.79	0.923	2.49	1.76	0.938	1.41	1.09	0.977	1.14	0.85	0.989	1.00	0.78	0.993
Mean, >50% clear sky	1.91	1.32	0.955	1.75	1.16	0.963	1.15	0.91	0.985	1.02	0.75	0.992	0.78	0.61	0.995
LLI(t), >50% clear sky	2.31	1.63	0.949	2.13	1.49	0.957	2.84	2.16	0.960	2.28	1.64	0.967	3.39	2.58	0.951
LAM_{PE}(t), >50% clear sky	2.63	1.91	0.938	3.62	2.53	0.941	3.19	2.43	0.943	4.05	2.64	0.853	4.08	2.97	0.870
LAM_{NE}(t), >50% clear sky	4.10	2.77	0.853	5.10	3.95	0.860	6.99	4.35	0.763	4.43	3.10	0.838	5.11	3.90	0.685
Mean, >50% clear sky	3.01	2.10	0.913	3.61	2.66	0.919	4.34	2.98	0.889	3.59	2.46	0.886	4.20	3.15	0.835
LLI(Q), >75% clear sky	1.05	0.76	0.990	0.67	0.48	0.995	0.84	0.70	0.994	0.58	0.45	0.998	0.45	0.37	0.999
LAM_{PE}(Q), >75% clear sky	1.29	0.88	0.982	1.56	0.96	0.967	0.73	0.59	0.995	0.79	0.57	0.995	0.58	0.48	0.998
LAM_{NE}(Q), >75% clear sky	1.77	1.31	0.970	1.43	0.99	0.979	0.98	0.77	0.991	0.79	0.60	0.995	0.72	0.55	0.995
Mean, >75% clear sky	1.37	0.98	0.980	1.22	0.81	0.980	0.85	0.69	0.994	0.72	0.54	0.996	0.58	0.47	0.997
LLI(t), >75% clear sky	1.93	1.39	0.966	1.15	0.89	0.990	2.24	1.65	0.973	1.46	0.90	0.982	2.70	1.93	0.989
LAM_{PE}(t), >75% clear sky	1.65	1.23	0.975	1.68	1.24	0.974	2.05	1.52	0.975	2.26	1.39	0.971	3.10	2.23	0.938
LAM_{NE}(t), >75% clear sky	3.77	2.50	0.880	3.86	2.91	0.922	8.16	5.24	0.764	3.63	2.73	0.940	3.40	2.76	0.933
Mean, >75% clear sky	2.45	1.70	0.940	2.23	1.68	0.962	4.15	2.80	0.904	2.45	1.67	0.964	3.06	2.31	0.953

Supplementary Table 4: Complete statistics for the validation on cloudy days when less than 50% of each degradation modes (sample size 11,000 points).

<50% degradation	RF			XGB			FNN			1DConv			DTW-CNN		
	RMSE (%)	MAE (%)	ρ	RMSE (%)	MAE (%)	ρ	RMSE (%)	MAE (%)	ρ	RMSE (%)	MAE (%)	ρ	RMSE (%)	MAE (%)	ρ
**LLI(Q), all days**	4.93	3.14	0.893	5.06	3.14	0.914	4.00	2.69	0.920	4.50	2.74	0.917	2.96	1.78	0.974
**LAM_{PE}(Q), all days**	4.15	2.85	0.875	3.55	2.39	0.955	3.16	2.32	0.929	3.97	2.67	0.917	2.49	1.78	0.977
**LAM_{NE}(Q), all days**	5.60	3.79	0.870	5.91	3.91	0.875	4.60	3.22	0.903	4.10	2.63	0.918	3.27	2.20	0.966
**Mean, all days**	4.89	3.26	0.879	4.84	3.15	0.915	3.92	2.75	0.917	4.19	2.68	0.917	2.91	1.92	0.973
**LLI(t), all days**	5.53	3.62	0.916	6.01	3.62	0.905	6.28	4.21	0.925	6.16	3.98	0.920	6.01	4.44	0.924
**LAM_{PE}(t), all days**	4.75	3.37	0.931	4.47	3.19	0.953	5.20	3.82	0.949	10.75	6.34	0.685	7.86	5.32	0.844
**LAM_{NE}(t), all days**	7.24	5.19	0.850	8.25	6.36	0.869	14.11	9.23	0.740	8.85	6.05	0.807	9.13	6.83	0.767
**Mean, all days**	5.84	4.06	0.899	6.25	4.39	0.909	8.53	5.75	0.871	8.59	5.46	0.804	7.67	5.53	0.845
**LLI(Q), >50% clear sky**	3.11	1.80	0.974	3.01	1.62	0.975	2.33	1.40	0.985	2.55	1.33	0.985	2.04	1.06	0.988
**LAM_{PE}(Q), >50% clear sky**	1.93	1.28	0.988	1.85	1.14	0.990	1.30	0.97	0.995	1.45	0.93	0.994	1.08	0.77	0.996
**LAM_{NE}(Q), >50% clear sky**	3.71	2.34	0.958	4.26	2.48	0.953	2.42	1.62	0.982	2.13	1.27	0.989	1.88	1.15	0.990
**Mean, >50% clear sky**	2.91	1.81	0.973	3.04	1.74	0.973	2.02	1.33	0.987	2.04	1.18	0.989	1.67	0.99	0.991
**LLI(t), >50% clear sky**	4.72	2.80	0.934	5.08	2.75	0.931	5.19	3.37	0.952	4.70	2.83	0.956	5.24	3.88	0.942
**LAM_{PE}(t), >50% clear sky**	3.61	2.39	0.959	3.42	2.40	0.973	4.27	3.02	0.962	9.49	5.14	0.715	6.70	4.38	0.893
**LAM_{NE}(t), >50% clear sky**	5.67	3.71	0.914	6.48	4.60	0.923	13.94	8.63	0.758	7.23	4.60	0.864	8.06	5.68	0.806
**Mean, >50% clear sky**	4.67	2.96	0.936	4.99	3.25	0.942	7.80	5.01	0.891	7.14	4.19	0.845	6.67	4.65	0.880
**LLI(Q), >75% clear sky**	2.77	1.53	0.979	2.54	1.27	0.983	2.04	1.24	0.989	1.96	0.97	0.990	1.59	0.80	0.992
**LAM_{PE}(Q), >75% clear sky**	1.50	1.01	0.994	1.61	1.00	0.992	0.97	0.71	0.997	1.17	0.69	0.996	0.75	0.55	0.998
**LAM_{NE}(Q), >75% clear sky**	2.94	1.89	0.977	3.51	1.89	0.967	1.77	1.15	0.992	1.83	0.99	0.992	1.16	0.78	0.997
**Mean, >75% clear sky**	2.41	1.48	0.983	2.56	1.39	0.981	1.59	1.03	0.993	1.65	0.88	0.993	1.16	0.71	0.996
**LLI(t), >75% clear sky**	3.00	1.96	0.977	4.80	2.20	0.937	4.34	2.78	0.962	3.98	2.16	0.961	3.74	2.77	0.973
**LAM_{PE}(t), >75% clear sky**	2.01	1.37	0.990	1.70	1.24	0.993	3.26	2.27	0.986	5.93	2.86	0.906	5.32	3.32	0.945
**LAM_{NE}(t), >75% clear sky**	4.41	2.95	0.955	5.69	3.90	0.941	14.71	9.71	0.775	5.19	3.79	0.951	5.30	3.64	0.930
**Mean, >75% clear sky**	3.14	2.09	0.974	4.07	2.44	0.957	7.43	4.92	0.908	5.03	2.94	0.940	4.78	3.25	0.950
