# Supplementary material for: Data-driven direct diagnosis of Li-ion batteries connected to photovoltaics
Source: Nat Commun. 2023 May 30;14:3138. doi: 10.1038/s41467-023-38895-7 (PMC10229535; doi:10.1038/s41467-023-38895-7)
Supplement: Supplementary file 2 — Reporting Summary [file 41467_2023_38895_MOESM2_ESM.pdf]

nature portfolio

Corresponding author(s): Matthieu Dubarry

Last updated by author(s): Jan 23, 2023

Reporting Summary

Nature Portfolio wishes to improve the reproducibility of the work that we publish. This form provides structure for consistency and transparency in reporting. For further information on Nature Portfolio policies, see our Editorial Policies and the Editorial Policy Checklist.

Statistics

For all statistical analyses, confirm that the following items are present in the figure legend, table legend, main text, or Methods section.

n/a	Confirmed	
☐	☒	The exact sample size (*n*) for each experimental group/condition, given as a discrete number and unit of measurement
☒	☐	A statement on whether measurements were taken from distinct samples or whether the same sample was measured repeatedly
☒	☐	The statistical test(s) used AND whether they are one- or two-sided<br>*Only common tests should be described solely by name; describe more complex techniques in the Methods section.*
☒	☐	A description of all covariates tested
☒	☐	A description of any assumptions or corrections, such as tests of normality and adjustment for multiple comparisons
☒	☐	A full description of the statistical parameters including central tendency (e.g. means) or other basic estimates (e.g. regression coefficient) AND variation (e.g. standard deviation) or associated estimates of uncertainty (e.g. confidence intervals)
☒	☐	For null hypothesis testing, the test statistic (e.g. *F*, *t*, *r*) with confidence intervals, effect sizes, degrees of freedom and *P* value noted<br>*Give P values as exact values whenever suitable.*
☒	☐	For Bayesian analysis, information on the choice of priors and Markov chain Monte Carlo settings
☒	☐	For hierarchical and complex designs, identification of the appropriate level for tests and full reporting of outcomes
☐	☒	Estimates of effect sizes (e.g. Cohen's *d*, Pearson's *r*), indicating how they were calculated

Our web collection on statistics for biologists contains articles on many of the points above.

Software and code

Policy information about availability of computer code

Data collection	Battery data collection was done using a Bio-Logic VMP3. Solar data collection was done using a Kipp & Zonen SMP21-A secondary standard pyranometer.
Data analysis	The battery digital twin was generated using the 2022 version of the 'alawa toolbox running under MatLab 2021. ML training and validation was done using custom python code available in https://github.com/NahuelCostaCortez/PVDiagnosis.

For manuscripts utilizing custom algorithms or software that are central to the research but not yet described in published literature, software must be made available to editors and reviewers. We strongly encourage code deposition in a community repository (e.g. GitHub). See the Nature Portfolio guidelines for submitting code & software for further information.

Data

Policy information about availability of data

All manuscripts must include a data availability statement. This statement should provide the following information, where applicable:

- Accession codes, unique identifiers, or web links for publicly available datasets
- A description of any restrictions on data availability
- For clinical datasets or third party data, please ensure that the statement adheres to our policy

All the sharable data from this work is available with the actual PV data and the synthetic cycles for perfect irradiance and cloud coverage.
Dubarry, M.; Costa, N.; Matthews, D. GIC//NMC Solar Battery Synthetic Data 1 - 700,000 degradation for 03/21 clear-sky irradiance. 2022.

Dubarry, M.; Costa, N.; Matthews, D. GIC//NMC Solar Battery Synthetic Data 2 - 45,000 x 18 degradation for clear-sky irradiance and cloudy days. 2022.

Human research participants

Policy information about studies involving human research participants and Sex and Gender in Research.

Reporting on sex and gender	N/A
Population characteristics	N/A
Recruitment	N/A
Ethics oversight	N/A

Note that full information on the approval of the study protocol must also be provided in the manuscript.

Field-specific reporting

Please select the one below that is the best fit for your research. If you are not sure, read the appropriate sections before making your selection.

☒ Life sciences ☐ Behavioural & social sciences ☐ Ecological, evolutionary & environmental sciences

For a reference copy of the document with all sections, see nature.com/documents/nr-reporting-summary-flat.pdf

Life sciences study design

All studies must disclose on these points even when the disclosure is negative.

Sample size	Since the data used in this work is synthetic, sample size was chosen according to calculation time.
Data exclusions	No data was excluded
Replication	A cell-to-cell variation study was undertaken by reproducing part of the simulations. Cell-to-cell variations were found to be negligible with on average 0.4% MAE with a 0.7% standard deviation between the two sets
Randomization	Synthetic data, no randomization was needed.
Blinding	Synthetic data, no blinding was needed.

Reporting for specific materials, systems and methods

We require information from authors about some types of materials, experimental systems and methods used in many studies. Here, indicate whether each material, system or method listed is relevant to your study. If you are not sure if a list item applies to your research, read the appropriate section before selecting a response.

Materials & experimental systems

n/a	Involved in the study	
☒	☐	Antibodies
☒	☐	Eukaryotic cell lines
☒	☐	Palaeontology and archaeology
☒	☐	Animals and other organisms
☒	☐	Clinical data
☒	☐	Dual use research of concern

Methods

n/a	Involved in the study	
☒	☐	ChIP-seq
☒	☐	Flow cytometry
☒	☐	MRI-based neuroimaging
