# Supplementary material for: Data-driven direct diagnosis of Li-ion batteries connected to photovoltaics
Source: Nat Commun. 2023 May 30;14:3138. doi: 10.1038/s41467-023-38895-7 (PMC10229535; doi:10.1038/s41467-023-38895-7)
Supplement: Supplementary file 3 — Solar Cells Reporting Summary [file 41467_2023_38895_MOESM3_ESM.pdf]

natureresearch

Corresponding author(s): Matthieu Dubarry

Solar Cells Reporting Summary

Nature Research wishes to improve the reproducibility of the work that we publish. This form is intended for publication with all accepted papers reporting the characterization of photovoltaic devices and provides structure for consistency and transparency in reporting. Some list items might not apply to an individual manuscript, but all fields must be completed for clarity.

For further information on Nature Research policies, including our data availability policy, see Authors & Referees.

Experimental design

Please check: are the following details reported in the manuscript?

1. Dimensions

Item	Yes/No	Details
Area of the tested solar cells	☐ Yes ☒ No	We only used the plane of array irradiance data of commercial systems
Method used to determine the device area	☐ Yes ☒ No	N/A

2. Current-voltage characterization

Item	Yes/No	Details
Current density-voltage (J-V) plots in both forward and backward direction	☐ Yes ☒ No	We only used the plane of array irradiance data of commercial system
Voltage scan conditions *For instance: scan direction, speed, dwell times*	☐ Yes ☒ No	We only used the plane of array irradiance data of commercial system
Test environment *For instance: characterization temperature, in air or in glove box*	☐ Yes ☒ No	Deployed commercial system
Protocol for preconditioning of the device before its characterization	☐ Yes ☒ No	Deployed commercial system
Stability of the J-V characteristic *Verified with time evolution of the maximum power point or with the photocurrent at maximum power point; see ref. 7 for details.*	☐ Yes ☒ No	Deployed commercial system

3. Hysteresis or any other unusual behaviour

Item	Yes/No	Details
Description of the unusual behaviour observed during the characterization	☐ Yes ☒ No	Deployed commercial system
Related experimental data	☐ Yes ☒ No	Deployed commercial system

4. Efficiency

Item	Yes/No	Details
External quantum efficiency (EQE) or incident photons to current efficiency (IPCE)	☐ Yes ☒ No	We only used the plane of array irradiance data of commercial system
A comparison between the integrated response under the standard reference spectrum and the response measure under the simulator	☐ Yes ☒ No	We only used the plane of array irradiance data of commercial system
For tandem solar cells, the bias illumination and bias voltage used for each subcell	☐ Yes ☒ No	We only used the plane of array irradiance data of commercial system

5. Calibration

Item	Yes/No	Details
Light source and reference cell or sensor used for the characterization	☐ Yes ☒ No	We only used the plane of array irradiance data of commercial system
Confirmation that the reference cell was calibrated and certified	☐ Yes ☒ No	We only used the plane of array irradiance data of commercial system

Calculation of spectral mismatch between the reference cell and the devices under test

- [ ] Yes
- [x] No

We only used the plane of array irradiance data of commercial system

6. Mask/aperture

Size of the mask/aperture used during testing

- [ ] Yes
- [x] No

We only used the plane of array irradiance data of commercial system

Variation of the measured short-circuit current density with the mask/aperture area

- [ ] Yes
- [x] No

We only used the plane of array irradiance data of commercial system

7. Performance certification

Identity of the independent certification laboratory that confirmed the photovoltaic performance

- [ ] Yes
- [x] No

We only used the plane of array irradiance data of commercial system

A copy of any certificate(s)
Provide in Supplementary Information

- [ ] Yes
- [x] No

We only used the plane of array irradiance data of commercial system

8. Statistics

Number of solar cells tested

- [ ] Yes
- [x] No

We only used the plane of array irradiance data of commercial system

Statistical analysis of the device performance

- [ ] Yes
- [x] No

We only used the plane of array irradiance data of commercial system

9. Long-term stability analysis

Type of analysis, bias conditions and environmental conditions
For instance: illumination type, temperature, atmosphere humidity, encapsulation method, preconditioning temperature

- [ ] Yes
- [x] No

We only used the plane of array irradiance data of a commercial system
